# CpxA Phosphatase Inhibitor Activates CpxRA and Is a Potential Treatment for Uropathogenic Escherichia coli in a Murine Model of Infection

**DOI:** 10.1128/spectrum.02430-21

**Published:** 2022-03-17

**Authors:** Kate R. Fortney, Sara N. Smith, Julia J. van Rensburg, Julie A. Brothwell, Jessi J. Gardner, Barry P. Katz, Nagib Ahsan, Adam S. Duerfeldt, Harry L. T. Mobley, Stanley M. Spinola

**Affiliations:** a Department of Microbiology and Immunology, Indiana University School of Medicine, Indianapolis, Indiana, USA; b Department of Biostatistics, Indiana University School of Medicine, Indianapolis, Indiana, USA; c Department of Medicine, Indiana University School of Medicine, Indianapolis, Indiana, USA; d Department of Pathology and Laboratory Medicine, Indiana University School of Medicine, Indianapolis, Indiana, USA; e Department of Microbiology and Immunology, University of Michigan Medical School, Ann Arbor, Michigan, USA; f Department of Chemistry and Biochemistry, University of Oklahoma, Norman, Oklahoma, USA; g Mass Spectrometry, Proteomics and Metabolomics Core Facility, Stephenson Life Sciences Research Center, University of Oklahoma, Norman, Oklahoma, USA; h Department of Biostatistics, Richard M. Fairbanks School of Public Health, Indiana University, Indianapolis, Indiana, USA; i Department of Medicinal Chemistry, University of Minnesota, Minneapolis, Minnesota, USA; University of Texas Southwestern Medical Center

**Keywords:** *Escherichia coli*, UPEC, CpxRA, phosphatase inhibitor, treatment

## Abstract

CpxRA is an envelope stress response system that is highly conserved in the *Enterobacteriaceae*. CpxA has kinase activity for CpxR and phosphatase activity for phospho-CpxR (CpxR-P), a transcription factor. In response to membrane stress, CpxR-P is produced and upregulates genes involved in membrane repair and downregulates genes that encode virulence factors that are trafficked across the cell membrane. Mutants that constitutively activate CpxRA in Salmonella enterica serovar Typhimurium and in uropathogenic Escherichia coli (UPEC) are attenuated in murine models. We hypothesized that pharmacologic activation of CpxR could serve as an antimicrobial/antivirulence strategy and recently showed that 2,3,4,9-tetrahydro-1*H*-carbazol-1-amines activate the CpxRA system by inhibiting CpxA phosphatase activity. Here, we tested the ability of a series of three CpxRA-activating compounds with increasing potency to clear UPEC stain CFT073 in a murine urinary tract infection model. We show that these compounds are well tolerated and achieve sufficient levels to activate CpxR in the kidneys, bladder, and urine. Although the first two compounds were ineffective in promoting clearance of CFT073 in the murine model, the most potent derivative, compound 26, significantly reduced bacterial recovery in the urine and trended toward reducing bacterial recovery in the bladder and kidneys, with efficacy similar to ciprofloxacin. Treatment of CFT073 cultured in human urine with compound 26 fostered accumulation of CpxR-P and decreased the expression of proteins involved in siderophore biosynthesis and binding, heme degradation, and flagellar movement. These studies suggest that chemical activation of CpxRA may present a viable strategy for treating infections due to UPEC.

**IMPORTANCE** The increasing prevalence of urinary tract infections (UTIs) due to antibiotic-resistant uropathogenic Escherichia coli (UPEC) is a major public health concern. Bacteria contain proteins that sense their environment and have no human homologs and, thus, are attractive drug targets. CpxRA is a conserved sensing system whose function is to reduce stress in the bacterial cell membrane; activation of CpxRA reduces the expression of virulence determinants, which must cross the cell membrane to reach the bacterial surface. We previously identified a class of compounds that activate CpxRA. We show in a mouse UTI model that our most potent compound significantly reduced recovery of UPEC in the urine, trended toward reducing bacterial recovery in the bladder and kidneys, did not kill UPEC, and downregulated multiple proteins involved in UPEC virulence. Since these compounds do not act by a killing mechanism, they have potential to treat UTIs caused by antibiotic-resistant bacteria.

## INTRODUCTION

Urinary tract infections (UTIs) afflict nearly half of all women in the United States at an estimated cost of $3.5 to $5 billion per year (1–3). Uropathogenic Escherichia coli (UPEC) causes approximately 85% of uncomplicated UTIs. Unfortunately, some UTIs caused by E. coli are difficult to treat due to bacterial expression of extended-spectrum β-lactamases and quinolone resistance (3–6) or are virtually untreatable due to acquisition of factors that encode carbapenem and colistin resistance (2, 3, 7–10).

Most antibiotics were discovered from libraries of natural products and synthetic compounds based on their ability to inhibit bacterial growth (11). In the past 50 years, this strategy has failed to identify new drug classes or targets for Gram-negative bacteria (11–13). The increasing incidence of infections caused by drug-resistant bacteria has prompted an urgent need for novel antimicrobial strategies (3).

One alternative strategy relies on identifying compounds that interfere with bacterial virulence factors and render organisms more susceptible to clearance by the host immune system (2, 3, 14–16). As a regulator of virulence-associated genes, the CpxRA two-component signal transduction system (2CSTS) is an attractive target for antimicrobial therapies. CpxRA allows Gram-negative bacteria to sense and respond to envelope stress (17–19). CpxA is an inner membrane sensor kinase/phosphatase, and CpxR is a response regulator. CpxRA is highly conserved in the *Enterobacteriaceae*, has no mammalian homologs, and targets different amino acids for phosphorelay than mammalian kinases and phosphatases (18, 20).

In response to envelope stress, CpxA autophosphorylates on a conserved histidine residue and transfers its phosphate group to a conserved aspartate residue in CpxR, activating the system (20). In the absence of membrane stress, CpxA acts as a net phosphatase, keeping CpxR dephosphorylated and inactive (17). When E. coli is cultured in minimal medium containing glucose, small molecule donors such as acetyl phosphate can transfer phosphate groups to CpxR (21, 22). Under such conditions, a *cpxA* deletion mutant, which lacks phosphatase activity, accumulates phosphorylated CpxR (CpxR-P) (20). In E. coli and other Gram-negative pathogens, CpxR-P downregulates the transcription of secreted or envelope-localized proteins, including several virulence determinants, likely in an attempt to relieve envelope stress (20, 23, 24). Uncontrolled genetic activation of CpxRA renders Salmonella enterica serovar Typhimurium avirulent in mice (25). For Haemophilus ducreyi, deletion of *cpxA* reduces the expression of seven virulence factors required for human infection and abolishes the ability of the organism to infect the skin of human volunteers (26–28). Similarly, colonization of the murine female lower genital tract is also impaired in a *cpxA* (*misS*) insertion mutant of Neisseria gonorrhoeae (29).

Comparison of the transcriptomes of UPEC strain CFT073Δ*cpxA*::*cat* to CFT073 showed that activation of CpxR downregulated multiple fitness and virulence genes and operons, including those that encode for the type 1 fimbriae, P pili, and F1C fimbriae adhesins and those involved in sensing and responding to phosphate limitation and iron and nickel transport and metabolism (30). Competition experiments in a female CBA/J murine UTI model showed that both CFT073Δ*cpxA*::*cat* and CFT073Δ*cpxR*::*cat* were outcompeted by CFT073 in the urine, bladder, and kidneys (30). However, in single-strain infection experiments, the *cpxR* mutant was recovered from the urine, bladder, and kidneys in amounts similar to the wild type, while the *cpxA* mutant was recovered at significantly lower levels than the wild type in the kidneys but not in the bladder or urine (30), indicating that activation of CpxR impairs UPEC persistence in the kidney. This study suggested that pharmacological activation of CpxR could have some utility in treating chronic kidney infections due to UPEC.

**FIG 1:**
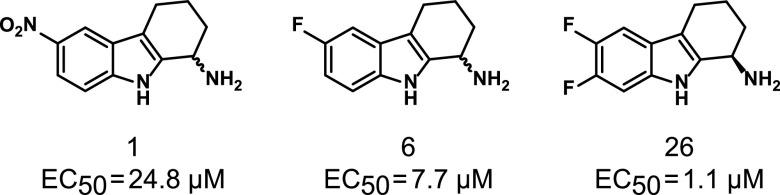
Structures of the compounds 1, 6, and 26 and their reported 50% effective concentration (EC_50_) in the Escherichia coli CpxR β-galactosidase reporter assay.

Previously, we developed a high-throughput screen in E. coli K-12 and identified one class of compounds that activate CpxR by inhibiting CpxA phosphatase activity (31). The initial hit, compound 1, a 6-nitro-2,3,4,9-tetrahydro-1*H*-carbazol-1-amine (Fig. 1), contained both a nitro group and primary amine, which were found to be dispensable and required for activity, respectively (31). Addition of electron-withdrawing groups on ring A increased compound potency (32). Compound 1 and its monofluoro (compound 6) and enantiomerically pure difluoro (compound 26) derivatives had mean 50% effective concentration (EC_50_) values of 24.8, 7.7, and 1.1 μM, respectively, in a β-galactosidase reporter assay for CpxRA activation in E. coli (Fig. 1) and increased the accumulation of CpxR-P as assessed by Phos-Tag gel electrophoresis (32).

As chemical induction of CpxR-P could result in diminished secretion of virulence determinants leading to clearance of UPEC by the host immune system, here we tested compound 1 and its derivatives for their ability to clear CFT073 in a murine UTI model. We report the safety and efficacy of compound 1; the safety, pharmacokinetic (PK) profile, and efficacy of compound 6; and the efficacy and mechanism of action of compound 26. We show that pharmacological activation of CpxRA is a viable strategy for treating UTIs caused by UPEC.

## RESULTS

### Compound 1 has no effect on CFU recovered in the murine UTI model.

We tested our initial hit, compound 1 (31) (Fig. 1), for toxicity in a murine model using criteria described in Materials and Methods. Except for decreased activity lasting 20 min after each dose, female CBA/J mice (*n* = 3) treated with 100 mg of compound 1/kg of body weight dissolved in 20% dimethyl sulfoxide (DMSO) and 40% glycerol in sterile water (hereafter called the vehicle control) subcutaneously (s.c.) twice a day for 3 days exhibited no toxicity; mice (*n* = 2) who received the vehicle control exhibited no toxicity. To test compound 1 for efficacy, female CBA/J mice were transurethrally inoculated with ∼1 × 10^8^ CFU of UPEC strain CFT073; 12 h later, mice were treated with 100 mg/kg compound 1 (*n* = 10) or the vehicle control (*n* = 5) s.c. twice a day for 3 days and then sacrificed 4 h after the last dose. No effect was seen on the recovery of E. coli CFT073 in the urine, bladder, or kidneys (see Fig. S1 in the supplemental material). As compound 1 lacked efficacy, we aimed to improve the scaffold to enhance potency and eliminate the nitro group liability (32).

### Compound 6 lacks acute toxicity, has favorable PK profiles, but lacks efficacy in the murine UTI model.

Substitution of the nitro group on compound 1 with a fluoride group yielded compound 6 (Fig. 1), whose mean EC_50_ value was 7.7 μM, which was 3.2-fold lower than that of compound 1 (32), and whose concentration for peak activation of CpxR was 10 μM (see Fig. S2 in the supplemental material). The activity of compound 6 in the reporter assay was not affected by 10% human AB serum (Fig. S2A). As measured in a lactate dehydrogenase (LDH) release assay (31), the 50% inhibitory concentration (IC_50_) for compound 6 after 5 h of incubation with HepG2 cells could not be calculated, as cell death was only observed at 2 concentrations; the calculated IC_50_ after 24 h of incubation was >33 μM (Fig. S2B).

**FIG 2:**
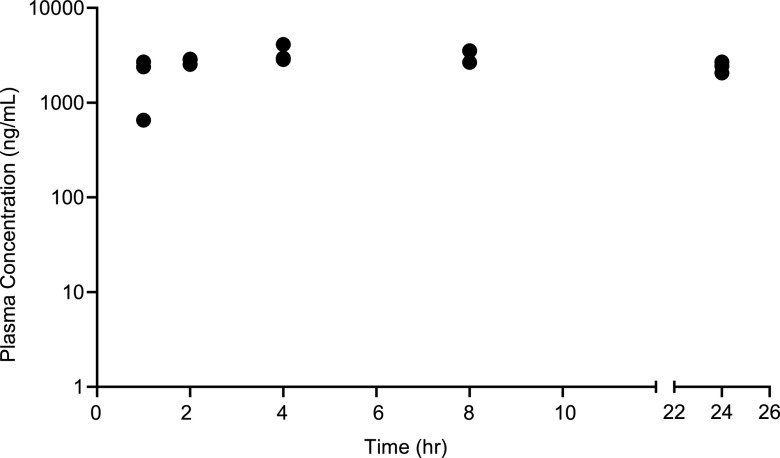
Single-dose pharmacokinetic parameters determined in female CBA/J mice (*n* = 15) given 100 mg/kg of compound 6 subcutaneously. Blood was obtained from groups of 3 mice 1, 2, 4, 8, and 24 h postinjection. Each symbol represents the plasma concentration of compound 6 obtained from 1 mouse.

We developed a high-performance liquid chromatography-tandem mass spectrometry (HPLC-MS/MS) assay to measure compound 6 with a lower limit of quantification of 1 ng/mL. Single-dose PK parameters of compound 6 were determined in female CBA/J mice (*n* = 15) given 100 mg/kg s.c. dissolved in the vehicle. Blood was obtained from groups of 3 mice 1, 2, 4, 8, and 24 h postinjection. After 4 h, compound 6 reached a mean ± standard deviation (SD) peak plasma concentration of 3,305 ± 696 ng/mL (16.2 ± 3.4 μM) and had a mean ± SD plasma concentration of 2,394 ± 316 ng/mL (11.7 ± 1.5 μM) at 24 h (Fig. 2). All compound-treated mice had slightly decreased activity and squinting 10 min after injection, lasting to the 8-h time point. One mouse developed slight tremors after 30 min that increased to severe at 2 h. Six mice also developed slight tremors after 1 h. No mice showed clinical signs of toxicity at 24 h.

As effective antimicrobials must achieve concentrations that exceed their MICs, we next determined whether compound 6 achieved plasma, kidney, bladder, and urinary concentrations in excess of its peak activating concentration of 10 μM in the CpxR reporter assay. Since the single-dose PK study showed that the concentration of compound 6 in the plasma exceeded 10 μM after 24 h, female CBA/J mice were injected s.c. with 100 mg/kg compound 6 (*n* = 3) or the vehicle control (*n* = 2) once a day at time zero, 24, and 48 h. Blood and urine samples were obtained just prior to the 48-h injection and at 52 h, 4 h after the final dose; we were only able to recover 2 urine samples at those time points. At 52 h, the animals were sacrificed, and tissue levels of compound 6 were determined in the bladder and kidneys. The mean peak and trough levels of compound 6 were 18.6 ± 3.2 μM and 9.6 ± 1.9 μM in the plasma and 344 μM and 151.7 μM in the urine, respectively. The mean tissue levels of compound 6 in the bladder and kidneys were 203 ± 28 μM and 48 ± 4 μM, respectively. Thus, compound 6 achieved levels in the urine, bladder, and kidneys that were ∼5- to 34-fold higher than its peak activating concentration.

We had shown that activation of CpxR by deletion of *cpxA* had no effect on the recoverable CFU of the mutant in the urine or bladder but significantly decreased the recovery of the mutant compared to recovery of the wild type in the kidneys of CBA/J female 48 h after transurethral infection (30). We therefore evaluated the efficacy of compound 6 in the murine model in a 4-arm trial that included a group inoculated with CFT073 and treated with the vehicle control, a group inoculated with CFT073Δ*cpxA*::*cat* and treated with the vehicle control, a group inoculated with CFT073 and treated with compound 6, and a group inoculated with CFT073 and treated with ciprofloxacin.

Given the variance in the log-transformed CFU in the urine, bladder, and kidneys of UPEC-infected mice 48 h after inoculation (33), we calculated that inclusion of 16 mice in each of 4 groups had a 90% power to detect a 2-log reduction in CFU in each treatment group versus the sham-treated control group, using a Student's *t* test after adjustment for comparisons of multiple groups. Although the trial required a total of up to 64 animals, for logistical reasons, we initially performed the trial in 4 groups of 8 animals with stopping rules. After results were obtained from 32 animals, an interim analysis was done to see whether significance had already been achieved with compound treatment or whether infecting additional animals was unlikely to show a compound effect; in both instances, the trial would be halted.

Four groups of eight female CBA/J mice were transurethrally inoculated with a target dose of ∼10^8^ CFU of either CFT073 or CFT073Δ*cpxA*::*cat.* One group was treated with ciprofloxacin (2.5 mg/kg/dose dissolved in sterile distilled water) s.c. 12, 24, 36, 48, and 60 h after inoculation with CFT073 as described (31). The untreated CFT073 and CFT073Δ*cpxA*::*cat* inoculated groups were given the vehicle control s.c. at 12, 24, 36, 48, and 60 h after inoculation. The compound 6 treated group were given the drug (100 mg/kg) s.c. at 12, 36, and 60 h after inoculation with CFT073 and received the vehicle control s.c. at 24 and 48 h. The animals were sacrificed 4 h after the last treatment (64 h postinoculation) and CFU per milliliter or CFU per gram of tissue was determined in the urine, bladder, and kidneys.

**FIG 3:**
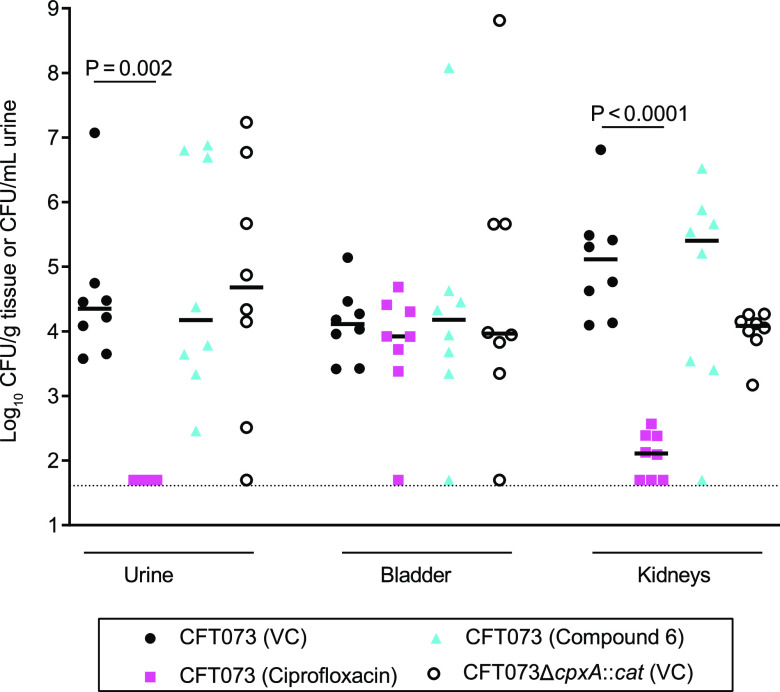
Recovery of bacteria from mice inoculated with CFT073 or CFT073Δ*cpxA*::*cat* and treated with the vehicle control (VC), ciprofloxacin, or compound 6 as described in text. The mice were sacrificed 4 h after the final treatment. The dashed line represents 50 CFU, the value assigned to samples with CFU below the limit of detection. After natural log transformation of the data, statistical comparisons were done using analysis of variance (ANOVA) with follow-up pairwise tests adjusted for multiple comparisons using the Tukey procedure. The recovery of CFT073 from ciprofloxacin-treated mice was significantly lower in the urine and kidneys than the vehicle control-treated mice. Otherwise, there were no significant differences among the groups.

Compared to mice inoculated with CFT073 and treated with the vehicle control, ciprofloxacin significantly reduced the CFU of CFT073 in the urine (*P* = 0.002) and kidneys (*P* < 0.0001) but not in the bladder; the latter result was expected since in this model CFT073 forms biofilms and enters quiescent states that are not antibiotic susceptible in the bladder (34) (Fig. 3). In contrast to previous data (30), mice inoculated with CFT073Δ*cpxA*::*cat* and treated with the vehicle control had the same CFU recovery as the CFT073-inoculated and vehicle-treated mice in the urine, bladder, and kidneys (Fig. 3). Compared to the CFT073-inoculated and vehicle-treated control group, treatment with compound 6 had no effect on the recovery of CFT073 in the urine, bladder, and kidneys (Fig. 3). An interim analysis showed that infecting more animals would not show an effect of compound 6; therefore, we stopped the trial. None of the compound 6-treated animals exhibited decreased activity, tremors, or squinting during the trial. Thus, despite adequate tissue and urine levels, compound 6 lacked efficacy in this model.

### Compound 26 demonstrates potential efficacy for treatment of UTIs in the murine model.

**FIG 4:**
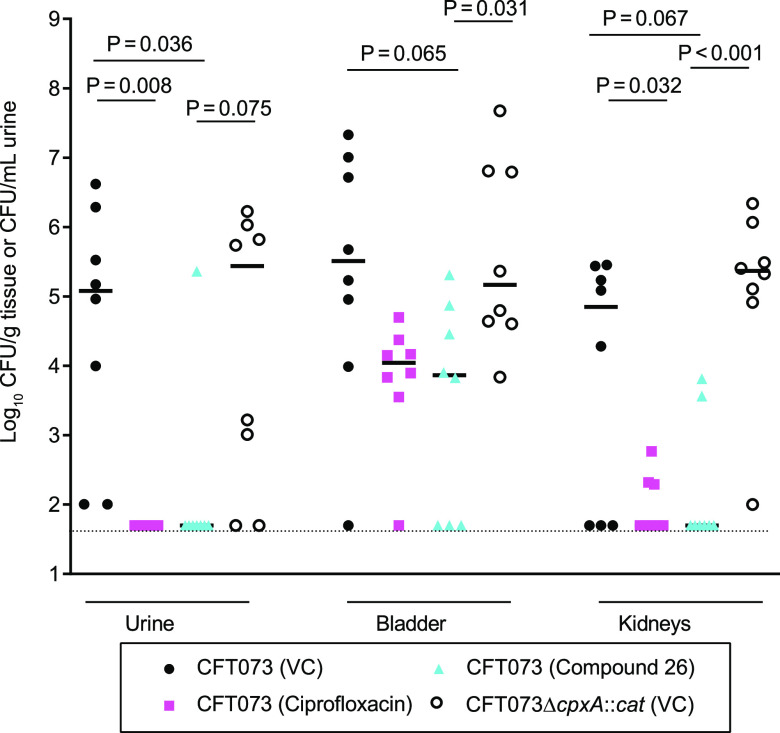
Recovery of bacteria from mice inoculated with CFT073 or CFT073Δ*cpxA*::*cat* and treated with the vehicle control (VC), ciprofloxacin, or compound 26 as described in text. The mice were sacrificed 4 h after the final treatment. The dashed line represents 50 CFU, the value assigned to samples with CFU below the limit of detection. Statistical comparisons were done as described in Fig. 3. The recovery of CFT073 from compound 26-treated mice was significantly reduced in the urine and trended toward being reduced in the bladder and kidneys compared to the vehicle control-treated and CFT073-inoculated mice. The recovery of CFT073 from compound 26-treated mice trended toward being reduced in the urine and was significantly reduced in the bladder and kidneys compared to the vehicle control-treated and CFT073Δ*cpxA*::*cat*-inoculated mice. The recovery of CFT073 from ciprofloxacin-treated mice was significantly lower in the urine and kidneys than the vehicle control-treated and CFT073-inoculated mice but was not different than the compound 26-treated mice in all 3 compartments.

After completing the compound 6 trial, we made an enantiomerically pure difluoro derivative of compound 1, compound 26 (Fig. 1), which had a mean EC_50_ value (1.1 μM) that was ∼7-fold lower than compound 6 (32). We evaluated the efficacy of compound 26 in the 4-armed trial using groups of eight mice exactly as described above. Mice inoculated with CFT073 and treated with ciprofloxacin had significantly lower recoverable CFU in the urine (*P* = 0.008) and kidneys (*P* = 0.032) but not the bladder (*P* = 0.174) than the CFT073-inoculated and vehicle-treated controls (Fig. 4). Treatment of mice inoculated with CFT073 with 100 mg/kg of compound 26 significantly lowered the recoverable CFU in the urine (*P* = 0.036) and trended toward lowering the CFU in the bladder (*P* = 0.065) and the kidneys (*P* = 0.067) compared to that of the CFT073-inoculated and vehicle-treated controls (Fig. 4). In comparing the groups inoculated with CFT073 and treated with compound 26 or ciprofloxacin, there were no significant differences in the recoverable CFU in the urine, bladder, and kidneys (all *P* > 0.9). Once again, the recoverable CFU from vehicle-treated mice inoculated with CFT073Δ*cpxA*::*cat* was not significantly different than the CFU recovered from vehicle-treated mice inoculated with CFT073 in the urine, bladder, and kidneys (Fig. 4). However, mice inoculated with CFT073 and treated with compound 26 had significantly lower recoverable CFU in the bladder (*P* = 0.031) and kidneys (*P* < 0.001) and trended toward having a lower CFU (*P* = 0.075) in the urine than vehicle-treated mice inoculated with CFT073Δ*cpxA*::*cat* (Fig. 4). As the interim analysis showed that treatment of animals with compound 26 had a significant effect on the recovery of CFT073 in the urine, we stopped the trial. None of the compound 26-treated animals exhibited signs of toxicity during the trial. These data indicate that chemical inhibition of CpxA phosphatase activity by compound 26 has efficacy similar to ciprofloxacin in the treatment of CFT073 in the murine model and that chemical inhibition of CpxA phosphatase activity has a greater effect than genetic deletion of *cpxA*, which removes both CpxA kinase and phosphatase activity. The data also suggest that CpxA kinase is active during infection.

### Compound 26 causes accumulation of CpxR-P in CFT073 cultured in human urine.

**FIG 5:**
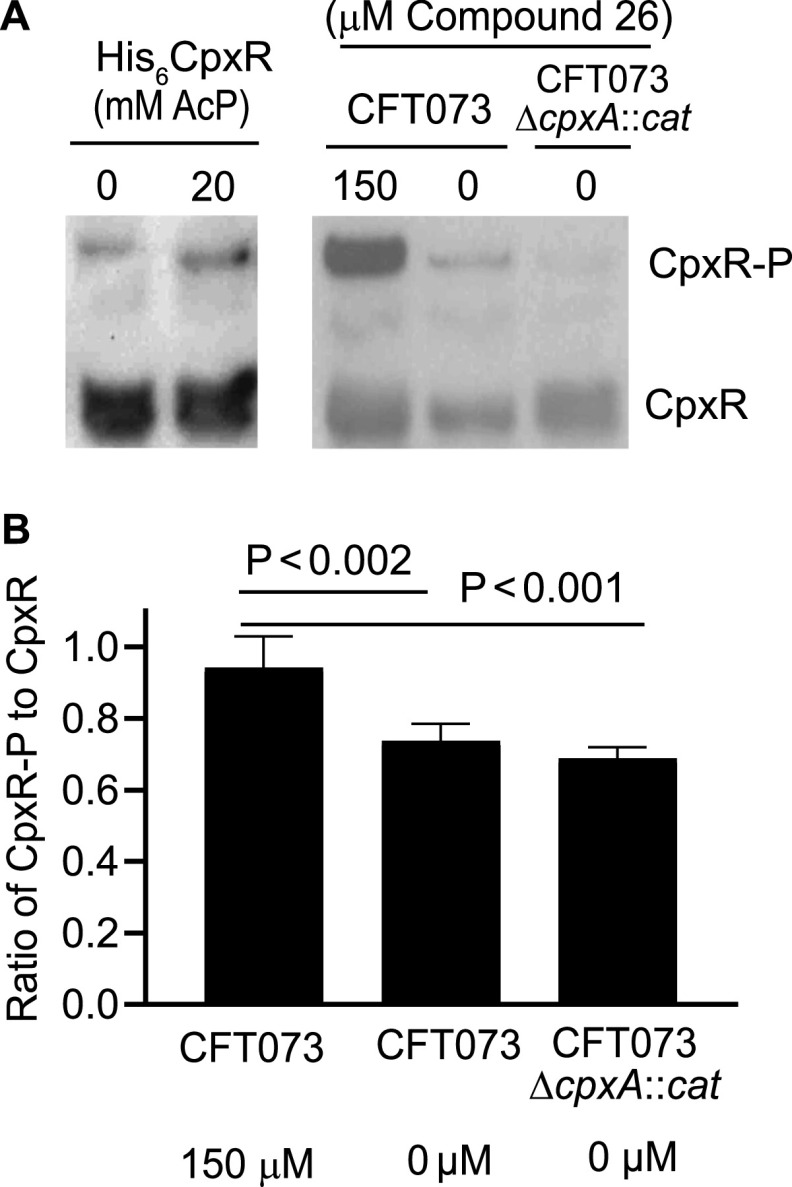
Compound 26 induces phospho-CpxR accumulation in CFT073 grown in human urine. (A) Composite representative Western blot of His_6_-CpxR incubated with 0 or 20 mM acetyl phosphate (AcP) (left) and CFT073 and the CFT073Δ*cpxA*::*cat* mutant grown in human urine with 0 or 150 μM compound (right). Note that migration of His_6_-CpxR is slower than native CpxR. (B) The ratio CpxR-P to CpxR was determined using densitometry; mean and standard deviation are from 4 independent experiments. *P* values were calculated with a one-way ANOVA adjusted for multiple comparisons using the Tukey procedure.

To examine whether treatment with compound 26 fostered higher accumulation of CpxR-P in CFT073 than in untreated CFT073 or CFT073Δ*cpxA*::*cat*, we cultured CFT073 in human urine in the presence of 150 μM compound 26 dissolved in DMSO, and CFT073 and CFT073Δ*cpxA*::*cat* in the presence of DMSO alone (hereafter called the CFT073 and CFT073Δ*cpxA*::*cat* controls); we chose 150 μM, as this was the approximate trough concentration of compound 6 detected in murine urine after 3 days of treatment. Briefly, the bacteria were grown in LB medium overnight, pelleted, washed, and inoculated into human urine and allowed to grow for 5 h. In 5 independent experiments, compound 26-treated CFT073 grew to a mean ± SD optical density at 600 nm (OD_600_) of 0.52 ± 0.2, while the CFT073 and CFT073Δ*cpxA*::*cat* controls grew to OD_600_ values of 0.41 ± 0.1 and 0.39 ± 0.03, respectively; these differences were not significant. We harvested bacterial cells, made cell lysates, and detected CpxR and CpxR-P by Western blotting with an anti-MBP-CpxR antiserum as described (31). As controls for the blots, we used recombinant CpxR that was untreated or treated with acetyl phosphate. Treatment of CFT073 with compound 26 induced accumulation of CpxR-P that was higher than that of the CFT073 and CFT073Δ*cpxA*::*cat* controls (Fig. 5). To compare the samples, the CpxR and CpxR-P bands were quantified by densitometry using samples from 4 of the independent experiments. Compound 26 increased the ratio of CpxR-P to CpxR in CFT073 by 1.3- (*P* < 0.002) and 1.4-fold (*P* < 0.001), respectively, compared to the CFT073 and CFT073Δ*cpxA*::*cat* controls. These data suggest that treatment of CFT073 by compound 26 does not inhibit bacterial growth and fosters accumulation of CpxR-P and that CpxA acts as a net kinase during growth in human urine.

### The proteome of compound 26-treated CFT073 grown in human urine is distinct from that of untreated CFT073Δ*cpxA*::*cat* and untreated CFT073.

**FIG 6:**
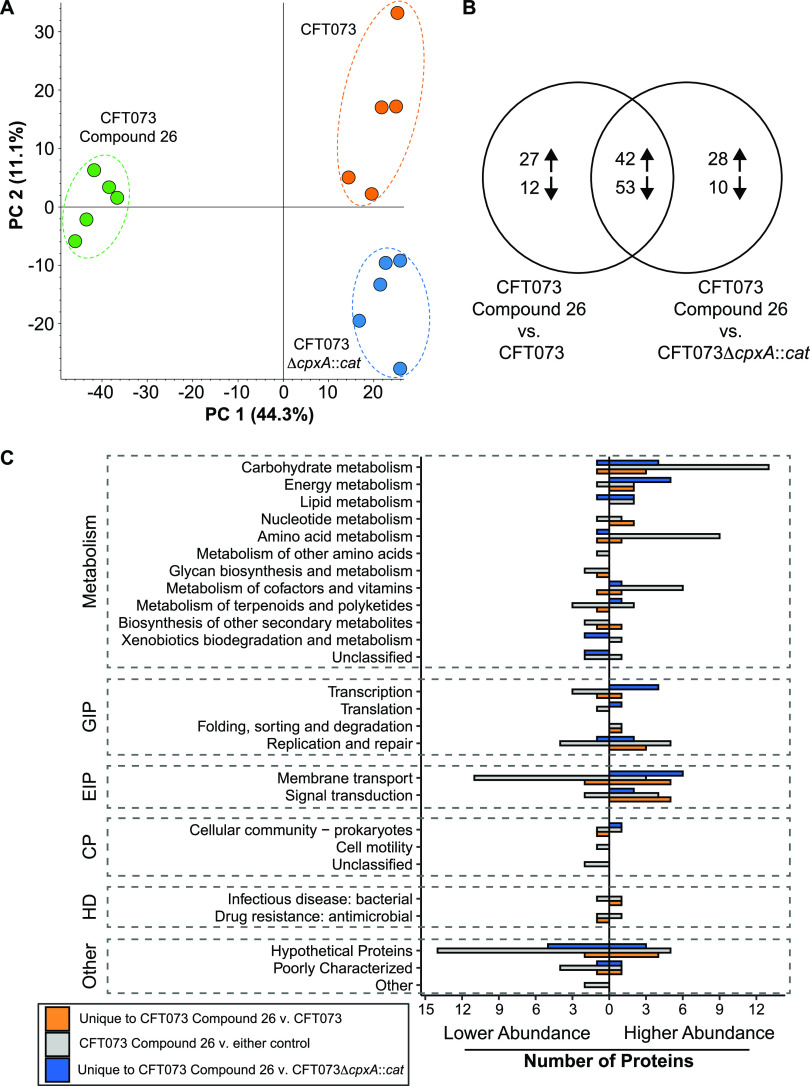
The proteome of compound 26-treated CFT073 differs from that of untreated CFT073 and CFT073Δ*cpxA*::*cat.* (A) Principal-component analysis of the proteomes of the three sample types from 5 independent experiments cluster distinctly. (B) Venn diagram showing the number of differentially abundant proteins between CFT073 treated with compound 26 versus either CFT073 or CFT073Δ*cpxA*::*cat* treated with DMSO. Higher- and lower-abundance proteins are indicated by up and down arrows, respectively. (C) Functional classification of proteins with 1.5-fold differential abundance at an adjusted *P* value of <0.05 following treatment of CFT073 with compound 26 versus the CFT073 or CFT073Δ*cpxA*::*cat* controls by KEGG identifiers. GIP, genetic information processing; EIP, environmental information processing; CP, cellular processes; HD, human diseases.

To explore the consequences of accumulation of CpxR-P in CFT073 treated with compound 26, the cell lysates collected after 5 h of growth in human urine were subjected to proteomic analysis. By principle component analysis, the proteomes of each of the three sample types (compound 26-treated CFT073 and the CFT073 and CFT073Δ*cpxA*::*cat* controls) from the 5 independent experiments formed distinct clusters (Fig. 6A). A label-free comparative quantitative analysis of the three sample types successfully identified and quantified a total of 2,241 proteins (see Table S1 in the supplemental material). Compared to both controls, CFT073 treated with compound 26 had 42 higher abundance proteins and 53 lower abundance proteins at a threshold of 1.5-fold with an adjusted *P* value of <0.05 (Fig. 6B). Compared to the CFT073 control, an additional 27 higher- and 12 lower-abundance proteins were present in CFT073 treated with compound 26; compared to the CFT073Δ*cpxA*::*cat* control, an additional 28 higher- and 10 lower-abundance proteins were present in CFT073 treated with compound 26 (Fig. 6B).

KEGG classifiers were used to categorize the differentially abundant proteins (Fig. 6C). The category with the greatest number of proteins was metabolism. Higher-abundance proteins expressed in CFT073 treated with compound 26 compared to both controls included those involved in transport (SapD and DppB) and metabolism (LeuCD, PutA, c1990, NadB, GltB, and IlvM) of peptides and amino acids, metal ion efflux/homeostasis (CusA and HydG), and capsule synthesis (KpsE) (see Table S2 in the supplemental material). Proteins that had higher abundance in CFT073 treated with compound 26 compared to the CFT073 control included additional peptide transporters (ArtM and TppB) and metabolizers of amino acids (DapB), proteins that provide resistance to various stressors (YkgD, CstA, YcgZ, YmbG, and Hnr), a protein known to enable the use of nitrate as a terminal electron receptor (NarY), and a protein that negatively regulates flagellum and biofilm formation (YbjN) (Table S2). Those with higher abundance in CFT073 treated with compound 26 compared to that of the CFT073Δ*cpxA*::*cat* control included proteins associated with the transport of peptides (DppF) and sugars (ExuT and AgaB), increased lipid biosynthesis and localization (c1200, c2468, and LptG), alterations in transcription (CspI, c3307, and c5025), a decrease in genome replication in nutrient stress (CspD), the use of formate as an electron donor (FdoH) and nitrate as an electron receptor (NarH) even in aerobic conditions (NuoN and NuoL), and uptake of ferrous iron (EfeU) (Table S2). There was also a substantial increase of an unnamed histidine kinase (c5041). Thus, treatment of CFT073 with compound 26 increases—rather than impairs—the production of several proteins involved in metabolism during growth in urine (35), consistent with its lack of inhibition of cell growth.

Proteins that were found in lower abundance in CFT073 treated with compound 26 than in both the CFT073 and CFT073Δ*cpxA*::*cat* controls included several involved in siderophore biosynthesis (EntB, EntF, YbdB, and IucB) and binding (FepB and c5174), metal ion transport (SitA, SitB, YohL, and ZntB), amino acid transport (BrnQ, YecS, and DsdX), flagellar movement (CheY and YojN), heme degradation (ChuS, ChuX, and ChuY), a transcription factor (YhiF) that regulates several stress response mechanisms (e.g., biofilm formation, acid resistance, and serum resistance), a cytopathic effector for bladder and kidney cells (SatE), and proteins that are involved in stress responses (YkgI, SodC, and YojN) (see Table S3 in the supplemental material). Additional proteins involved in siderophore synthesis (EntE and IucD) and iron acquisition (FepC and YcdO) and amino acid synthesis (c1220) were in lower abundance in CFT073 treated with compound 26 than in the CFT073 control (Table S3). Additional lower-abundance proteins in CFT073 treated with compound 26 compared to the CFT073Δ*cpxA*::*cat* control included those involved in iron release from siderophores (IroD) and modulators of general secretion (MsyB and HtpX) (Table S3). Taken together, the data suggest that treatment of CFT073 with compound 26 leads to decreases in at least 11 proteins involved in iron acquisition and utilization, which are critical for virulence and expressed during growth in human urine or in human UTIs (36–38). In addition, treatment with compound 26 decreased the abundance of proteins involved in flagellar motility, stress responses, and cytopathic effects, which may explain why treatment led to organism clearance in the urinary tract (36–38).

## DISCUSSION

The prevalence of UTIs in women and the increase in drug-resistant *Enterobacteriaceae* has prompted considerable interest in novel antimicrobial strategies. Targeting virulence mechanisms rather than survival processes may alleviate selective pressure on bacteria and result in decreased incidence of drug resistance. We and others have shown that genetic activation of the CpxRA 2CSTS downregulates the expression of key virulence factors and attenuates the virulence of bacteria from diverse species, including H. ducreyi, N. gonorrhoeae, *S.* Typhimurium, and UPEC in both human and animal models of infection (25, 26, 29, 30). To investigate the utility of CpxRA activation as an antimicrobial strategy, we identified and optimized a class of compounds that activate CpxR by inhibiting CpxA phosphatase activity (31, 32). Here, we show that this class of compounds is well-tolerated in mice and that our most potent derivative clears or tends to clear bacteria from the urine, bladder, and kidneys in a murine UTI model. These results suggest that CpxA phosphatase inhibitors may be useful in treating UTIs caused by UPEC.

We found that our original hit, compound 1, was ineffective against UPEC in the murine UTI model. We next evaluated a 6-fluoro analog of compound 1, compound 6, which is 3-fold more potent than compound 1 (32). Although in the dose used to treat infected animals, compound 6 exceeded its peak activating concentration in the urine, bladder, and kidneys, it had no efficacy in clearing UPEC from these compartments in mice. Although compound 6 lacked efficacy in the model, compound 26, which is 7-fold more potent than compound 6, caused significant reductions or trends toward reductions of CFT073 in the urine, the bladder, and kidneys compared to those in untreated mice inoculated with either CFT073 or CFT073Δ*cpxA*::*cat*. The reductions in recoverable CFU observed with compound 26 treatment were not significantly different than those observed following treatment with ciprofloxacin. Due to a lack of resources, we did not determine the urine or tissue levels of compound 26 in mice. Although there appears to be a relationship between compound potency and clearance of UPEC, we do not know if the efficacy of compound 26 was due to improved potency and/or pharmacokinetic parameters relative to the other compounds.

A major issue in the treatment of UTIs is the inability of antibiotics to clear bacteria from privileged compartments, such as the bladder and kidney, where bacterial persistence can lead to recurrent infections or renal scarring, especially in children. Ciprofloxacin, the antibiotic control used in these studies, reduced the recovery of CFT073 in the urine and kidneys but not in the bladder, which may be due to several factors, including biofilm formation, UPEC entry into a quiescent state within host cells, and the permeability barrier function of the uroepithelium (3, 34). Since compound 26 does not act by a killing mechanism, it could have had activity against bacteria that were not replicating in the bladder. Compared to untreated mice inoculated with CFT073, compound 26 trended to reduce bacterial recovery in both the bladder and kidney of CFT073 inoculated mice. Compared to untreated mice inoculated with CFT073Δ*cpxA*::*cat*, compound 26 significantly reduced bacterial recovery in both the bladder and kidney of CFT073 inoculated mice. Thus, this class of compounds may have the potential to reduce bacterial persistence in the bladder and kidney compartments.

We had previously shown that the CFT073Δ*cpxA*::*cat* was attenuated in the kidneys, but not in the bladder or urine, after 3 days of infection (30). Since the *cpxA* deletion mutant lacks both kinase and phosphatase activity, phosphorylation of CpxR in this strain requires bacterial metabolism of glucose into acetyl phosphate. As urine usually contains no glucose and the bladder epithelium does not store glucose, we had hypothesized that the attenuation of CFT073Δ*cpxA*::*cat* in the kidneys implied that the strain was utilizing glucose as a carbon source in this compartment. We had included CFT073Δ*cpxA*::*cat* as a positive control for the effect of CpxR activation in the murine model and had expected that the compounds would decrease the recovery of CFT073 only in the kidneys. Surprisingly, in this study, the CFT073Δ*cpxA*::*cat* was recovered in amounts similar to CFT073 in the urine, bladder, and kidneys in untreated mice; and treatment of mice with compound 26 significantly reduced or trended to reduce the recovery of CFT073 in all 3 compartments relative to that in untreated CFT073 or CFT073Δ*cpxA*::*cat*. The discrepancy in the behavior of CFT073Δ*cpxA*::*cat* in the two studies may be due to the stress induced by the injection of the vehicle control in this study. Alternatively, the mice in the previous study came from a different supplier (Envigo) than the mice used in the compound 6 and 26 trials (Jackson Laboratories); there is emerging literature about discrepancies in the virulence of pathogens in mice obtained from different vendors, perhaps due to differences in their gastrointestinal microbiomes (39, 40).

The fact that compound 26 decreased the recovery of CFT073 in the urine led us to hypothesize that CpxA acts as a net kinase *in vivo* and allowed the phosphatase inhibitor to foster accumulation of CpxR-P and downregulate virulence determinants. To test this hypothesis, we grew CFT073 in human urine in the presence and absence of compound 26 and found that treatment fostered accumulation of CpxR-P. Additionally, compound 26 treatment of CFT073 grown in urine caused more accumulation of CpxR-P than that of the CFT073Δ*cpxA*::*cat* control; this result is consistent with the fact that accumulation of CpxR-P in the mutant requires glucose, which is absent in urine. Lacking pharmacokinetic data for compound 26, a limitation of these experiments is that we used the concentration (150 μM) of compound 6 found in the urine after a 3-day dosing regimen.

To examine the effects of compound 26 on the production of virulence determinants, we compared the proteomes of CFT073 treated with the compound to the proteomes of untreated CFT073 and untreated CFT073Δ*cpxA*::*cat* grown in urine. We found that compound treatment caused decreased expression of several proteins involved in virulence, including those required for iron acquisition and metabolism, flagellar motion, and stress responses (36–38). This result was surprising in that transcriptome analysis of CFT073Δ*cpxA*::*cat* versus CFT073 grown to stationary phase in LB medium containing glucose indicated that genetic activation of CpxR fostered downregulation of expression of several genes in the type 1 fimbriae gene cluster, the *pstSCAB-phoU* operon and *phoBR* genes, which are important for sensing and responding to phosphate levels and positively regulate *fim* expression; the *cus* genes, which protect UPEC from copper toxicity; and nickel transport and metabolism genes (30). Transcriptome analysis did show downregulation of *tonB* and *chuA*, which are involved in iron metabolism and transport (30). Although the abundance of ChuA was not significantly altered in the proteome, ChuS, ChuX, and ChuY, which are encoded by genes in the same or neighboring operon, were significantly lower in abundance in compound 26-treated CFT073 than in the CFT073 or CFT073Δ*cpxA*::*cat* controls. Otherwise, there was little overlap between the differential expression of proteins between the compound 26-treated and untreated CFT073 and the differential expression of transcripts between CFT073Δ*cpxA*::*cat* and CFT073. In fact, opposite effects were found on the expression of *cusA* transcripts and its protein product in the two studies. This is likely due to the differences in growth conditions between the two studies and the multiple steps and regulatory mechanisms that take place between transcription and protein expression. In addition, CpxR was activated genetically in the transcriptome study and pharmacologically in this study; while genetic activation is unlikely to have off-target effects on other response regulators, pharmacological treatment may have such effects by inhibiting phosphatases of 2CSTS other than CpxA.

In conclusion, we have demonstrated that a CpxA phosphatase inhibitor has the potential to treat UPEC in a murine infection model. Although we have shown that these inhibitors activate CpxR, we do not know if they have off-target effects on other bacterial 2CSTS. Future studies will address the selectivity of the inhibitors and their pharmacokinetics. Given that E. coli CpxA and CpxR share 95% to 99% amino acid identity with homologs in Klebsiella, Enterobacter, Salmonella, and *Citrobacter* spp., future preclinical studies will also address whether the inhibitors can provide effective treatment for other antibiotic-resistant pathogens. In addition, whether these compounds reduce bacterial persistence in the bladder and kidney and their effect on commensals that contain CpxRA should be explored.

## MATERIALS AND METHODS

### Bacterial strains, mammalian cells, and growth conditions.

E. coli strain CFT073 and its derivative CFT073Δ*cpxA*::*cat* were described previously and maintained on Luria broth (LB) plates at 37°C (30). The E. coli *PcpxP′-lacZ* reporter strain, PAD282, was the gift of Thomas Silhavy (Princeton University, Princeton, NJ, USA) and was maintained on TB plates at 37°C (31). HepG2 hepatocellular carcinoma cells were the gift of Andy Yu (Indiana University, Indianapolis, IN) and were grown in RPMI 1600 medium (Gibco) containing 10% fetal calf serum (HyClone) and 1 mM sodium pyruvate (Sigma) at 37°C with 5% CO_2_ as described (31).

### Compounds and measurement of their EC_50_ values in a β-galactosidase assay.

Compounds 1 and 6 were purchased from ChemDiv and Aurum Pharmatech, respectively (31, 32). Compound 26 was synthesized and purified by the Duerfeldt laboratory as described (32). All compounds were dissolved in DMSO prior to use in this assay. The EC_50_ values for each compound were determined by measuring the β-galactosidase activity of the reporter strain PAD282 as previously reported (31). Briefly, PAD282 was cultured overnight in TB medium with 0.4% glucose and diluted into a 384-well plate, which contained the compounds in concentrations ranging from 0 to 160 μM, and incubated for 5 h at 37°C with no shaking. The All-in-One β-galactosidase reagent (Pierce) was added and the OD_420_, OD_550_, and OD_600_ of the wells were measured by a SpectraMax 384 plate reader. EC_50_ values were determined from 3 separate experiments done in triplicate using GraphPad Prism software using a nonlinear fit model. In some experiments, EC_50_ values were determined in the presence and absence of 10% human AB serum (Sigma).

### Cytotoxicity assay.

The cytotoxicity of compound 6 for HepG2 cells was determined by LDH release after incubation with the compound in concentrations ranging from 0 to 160 μM for 5 h and 24 h exactly as described (31). The IC_50_ was determined from quadruplicate experiments by the GraphPad Prism 6 software using a nonlinear fit model.

### Toxicology and PK studies.

Animal experiments were conducted according to the guidelines of the Indiana University School of Medicine Institutional Animal Care and Use Committee by the IU Simon Comprehensive Cancer Center In Vivo Therapeutics Core, a component of the Indiana Clinical Translational Science Institute. For the pilot toxicity study with compound 1, 4- to 6-week-old CBA/J female mice were obtained from Jackson Laboratory (Bar Harbor, ME) and were treated with compound 1 or the vehicle control twice a day for 3 days as described in the results. The mice were observed for 14 days. Toxicity was determined by the following criteria: deteriorating health-hunched posture, lack of grooming, failure to thrive, failure to eat and drink for >48 h, rapid loss of 15 to 20% body weight, inactivity with hunched posture, loss of righting reflex and inability to maintain upright posture, loss of general body condition with spine becoming visible, and dehiscence of wounds and/or evidence of infection at injection sites not responsive to veterinary treatment.

Compound 6 was quantified by the IU Simon Comprehensive Cancer Center Clinical Pharmacology Analytical Core from mouse plasma and mouse urine using temazepam (TMP) as the internal standard and HPLC-MS/MS (5500 QTRAP; AB Sciex, Framingham, MA). In brief, compound 6 and TMP were separated by a gradient mobile phase (acetonitrile, 5 mM ammonium acetate) with a Restek Ultra C_8_ 50 by 4.6 mm 5-micron column. The mass spectrometer utilized an electrospray ionization probe run in positive mode. The multiple reaction monitoring (MRM) Q1/Q3 (*m/z*) transitions for compound 6 and TMP were 187.9/146.0 and 301.1/254.9, respectively. Plasma or urine samples (20 μL) were transferred to glass culture tubes, TMP (20 μL of 0.1 ng/μL) was added as the internal standard, and the extraction was performed by the addition of 2 mL of methyl tertiary butyl ether. The samples were then vortexed, centrifuged, and the organic layer was transferred to a clean glass culture tube and evaporated to dryness. The samples were then reconstituted with 50 μL of the gradient mobile phase, and a 10-μL aliquot was injected to the HPLC-MS/MS. The standard curves were prepared using naive mouse plasma or naive mouse urine as the matched matrix and was linear from 1 to 1,000 ng/mL.

Compound 6 was quantified from the bladder and kidneys using a slightly modified method from the plasma sample analysis. Briefly, the tissue was weighed and then transferred to a polypropylene tube. Phosphate-buffered saline (PBS) (pH 7.4) was added to the tissue to bring the total volume to 0.5 mL (assumption 1 g = 1 mL). The tissue was homogenized using a TissueRuptor with a single use disposable probe. An aliquot (0.4 mL) was transferred to a clean polypropylene tube and temazepam (20 μL of 0.1 ng/μL) was added as the internal standard. The extraction procedure and HPLC-MS/MS conditions were the same as for the plasma samples. The standard curve was prepared using phosphate-buffered saline and was linear from 0.04 to 40 ng/sample.

### Murine infection experiments.

Animal experiments were conducted according to the guidelines of the University of Michigan Institutional Animal Care and Use Committee. For the animal infection experiments, bacterial strains were cultured at 37°C in LB medium overnight with shaking (30). Female CBA/J mice (Jackson Laboratories, Bar Harbor, ME) were inoculated transurethrally with a target dose of ∼10^8^ CFU of CFT073 or CFT073Δ*cpxA*::*cat.* Mice were subsequently treated 12 h later either with the vehicle, ciprofloxacin hydrochloride (PanReac AppliChem ITW Reagents; Jade Scientific), compound 1, compound 6, or compound 26 as described in Results and euthanized 4 h after the last treatment. Bacterial loads of each strain were calculated from the urine, bladder, and kidneys of mice as previously described; the limits of detection for these assays were 100 CFU/mL or 100 CFU/g of urine and tissue, respectively (33). Values of 50 CFU/mL or 50 CFU/g were assigned to specimens whose yields were below the limit of detection.

### Statistical analysis.

Due to the extreme skewness of the data, natural log transformation was used before the data were analyzed statistically. Descriptive statistics were calculated for all of the log-transformed variables. Statistical comparisons were done using analysis of variance (ANOVA) with follow-up pairwise tests adjusted for multiple comparisons using the Tukey procedure.

### Detection of CpxR-P and proteomics.

E. coli CFT073 and CFT073Δ*cpxA*::*cat* were cultured overnight at 37°C in 5 mL of LB medium overnight with shaking. Bacteria were pelleted, washed twice in PBS, and suspended in 1 mL of PBS. Cells were then suspended in 10 mL of filter-sterilized pooled human urine obtained from 4 healthy female volunteers as described (41) to an OD_600_ of ∼0.02. Urine containing CFT073 was treated either with 150 mM compound 26 dissolved in 10 μL of DMSO to reach a final concentration of 150 μM or 10 μL of DMSO; urine containing CFT073Δ*cpxA*::*cat* was treated with 10 μL of DMSO. After 5 h of incubation, cultures were split into 5-mL aliquots, centrifuged, and the pellets were washed once with PBS and frozen at −80°C. One pellet from 4 independent experiments was used to detect CpxR-P using a Phos-Tag gel and Western blotting with an anti-MBP-CpxR antibody obtained from Thomas Silhavy as previously described (31). Densitometry values were determined using Photoshop 2021 (version 22.4.3), and the ratio of CpxR-P to CpxR was analyzed by one-way ANOVA adjusted for multiple comparisons using the Tukey procedure.

For label-free quantitative proteomics, cell pellets from 5 independent experiments were subjected for protein extraction followed by liquid chromatography-tandem mass spectrometry (LC-MS/MS). Sample preparation for LC-MS/MS analysis and proteomics pipeline was followed as described previously (42). In brief, bacteria were pelleted from urine cultures, and the pellets were solubilized in 1 mL of 8 M urea lysis buffer. Equal amounts of proteins (100 μg) were subjected for overnight trypsin/LysC (V5071; Promega) digestion. One microgram of bovine serum albumin (BSA) was added to each sample as an internal control prior to in-solution digestion. LC-MS/MS analysis was performed on a Dionex UltiMate 3000 (Thermo Fisher Scientific) system connected to a Q-Exactive HF-X mass spectrometer (Thermo Fisher Scientific). Detail settings of the LC-MS/MS analysis was described previously (42).

Peptide spectrum matching of MS/MS spectra was searched against the UniProt E. coli CFT073-UPEC database (TaxID 199310) using the Sequest algorithm within Proteome Discoverer version 2.4 software (Thermo Fisher Scientific). The Sequest database search was performed with the following parameters: trypsin enzyme cleavage specificity, 2 possible missed cleavages, 10 ppm mass tolerance for precursor ions, 0.02 Da mass tolerance for fragment ions. Search parameters permitted dynamic modification of methionine oxidation (+15.9949 Da) and static modification of carbamidomethylation (+57.0215 Da) on cysteine. Peptide assignments from the database search were filtered to a 1% false discovery rate. The relative label-free quantitative and comparative among the samples were performed using the Minora algorithm and Proteome Discoverer 2.4 software. Differential abundance of proteins between conditions was defined as a 1.5-fold change in relative abundance with an adjusted *P* value of <0.05.

### Data availability.

The raw data from the proteomics experiments is deposited in MassIVE as MSV000088484.
