# Supplementary material for: CpxA Phosphatase Inhibitor Activates CpxRA and Is a Potential Treatment for Uropathogenic Escherichia coli in a Murine Model of Infection
Source: Microbiol Spectr. 2022 Mar 17;10(2):e02430-21. doi: 10.1128/spectrum.02430-21 (PMC9045377; doi:10.1128/spectrum.02430-21)
Supplement: SUPPLEMENTAL FILE 1 — Supplemental material. Download SPECTRUM02430-21_Supp_1_seq3.pdf, PDF file, 0.2 MB [file spectrum02430-21_supp_1_seq3.pdf]

**Figure S1**

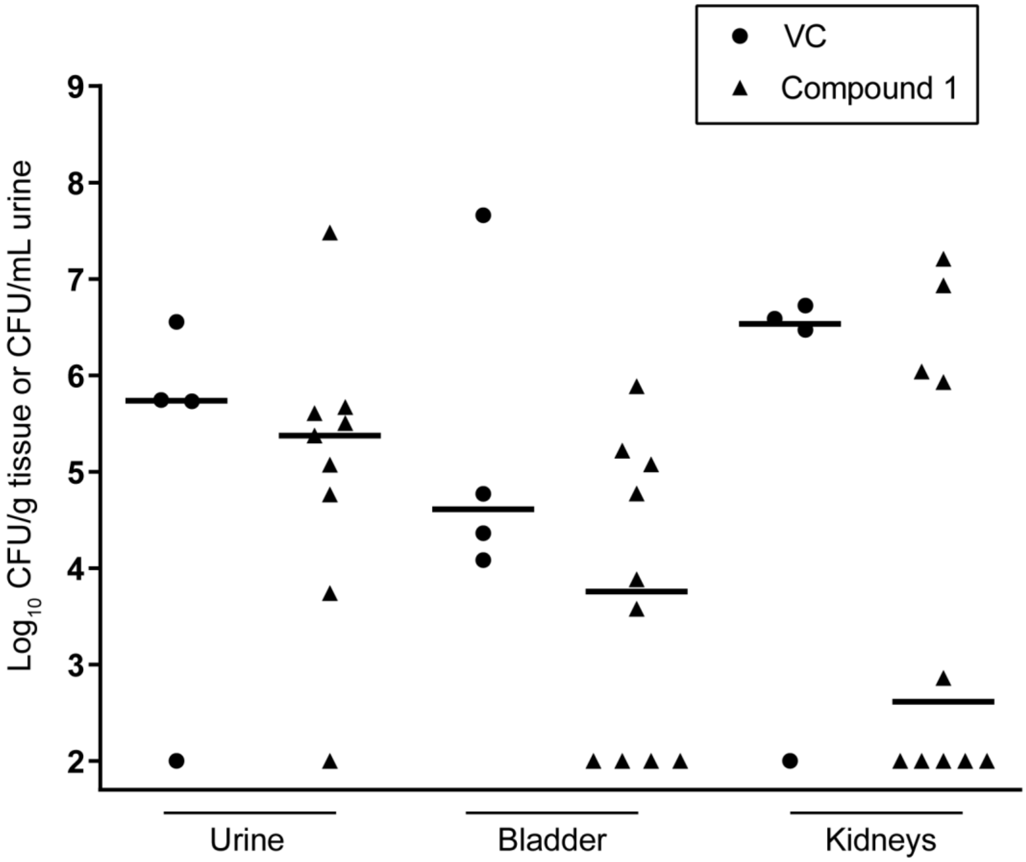


**FIG S1** Effect of compound 1 (100 mg/kg given subcutaneously twice a day for 3 days) beginning 12 hours after transurethral inoculation of ~1 x 108 CFU of CFT073 vs. the vehicle control (VC) given in the same schedule on the recovery of CFT073 in the urine, bladder, and kidneys. The animals were sacrificed 4 hours after the final dose. There were 10 animals in the compound 1 treated group and 5 in the VC treated group; one animal in the VC group died due to a bladder perforation 24 hours after inoculation. Statistical comparisons were done as described in Fig. 3. There were no significant differences between the groups.

**Figure S2**

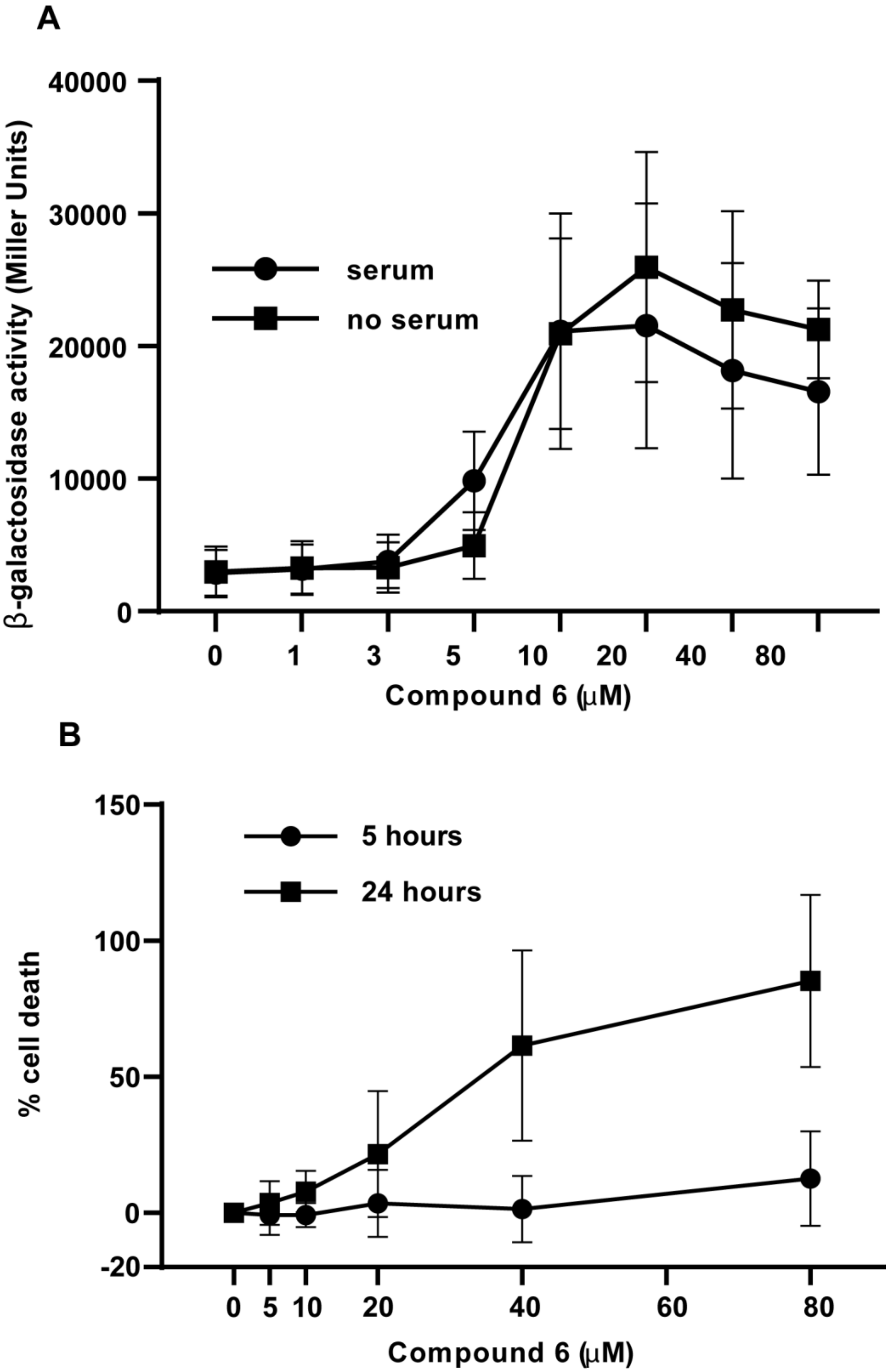


**FIG S2** Compound 6 activity in 10% serum and cytotoxicity with HepG2 cells. (A) *E. coli* CpxR-β-galactosidase reporter activity was measured in the presence (circles) or absence (squares) of 10% human AB serum. Note the peak activity of compound 6 is achieved at ~ 10 μM. The data are the mean ± SD from 3 independent experiments. (B) Cytotoxicity of compound 6 with HepG2 cells after treatment for 5 hours (squares) or 24 hours (circles). Cell viability was determined by LDH release. The data are representative of the mean ± SD from 4 independent experiments.
